# CDC25A^Q110del^: A Novel Cell Division Cycle 25A Isoform Aberrantly Expressed in Non-Small Cell Lung Cancer

**DOI:** 10.1371/journal.pone.0046464

**Published:** 2012-10-05

**Authors:** Rania H. Younis, Wei Cao, Ruxian Lin, Ronghui Xia, Zhenqiu Liu, Martin J. Edelman, Yuping Mei, Li Mao, Hening Ren

**Affiliations:** 1 Department of Oncology and Diagnostic Sciences, School of Dentistry, University of Maryland Baltimore, Baltimore, Maryland, United States of America; 2 Department of Oral and Maxillofacial Surgery, Jiao Tong University School of Stomatology, Shanghai, China; 3 Department of Oral Pathology, 9th People Hospital, Shanghai Jiao Tong University, School of Medicine, Key Laboratory of Stomatology, Shanghai, China; 4 Department of Epidemiology, School of Medicine, University of Maryland Baltimore, Baltimore, Maryland, United States of America; 5 University of Maryland Greenebaum Cancer Center, University of Maryland Baltimore, Baltimore, Maryland, United States of America; Virginia Commonwealth University, United States of America

## Abstract

**Objective:**

Lung cancer remains number one cause of cancer related deaths worldwide. Cell cycle deregulation plays a major role in the pathogenesis of Non-Small Cell Lung Cancer (NSCLC). CDC25A represents a critical cell cycle regulator that enhances cell cycle progression. In this study we aimed to investigate the role of a novel CDC25A transcriptional variant, CDC25A^Q110del^, on the regulation of the CDC25A protein, and its impact on prognosis of NSCLC patients.

**Methodology/Principal Findings:**

Here we report a novel CDC25A transcript variant with codon 110 (Glutamine) deletion, that we termed CDC25A^Q110del^ in NSCLC cells. In 9 (75%) of the 12 NSCLC cell lines, CDC25A^Q110del^ expression accounted for more than 20% of the CDC25A transcripts. Biological effects of CDC25A^Q110del^ were investigated in H1299 and HEK-293F cells using UV radiation, flowcytometry, cyclohexamide treatment, and confocal microscopy. Compared to CDC25A^wt^, CDC25A^Q110del^ protein had longer half-life; cells expressing CDC25A^Q110del^ were more resistant to UV irradiation and showed more mitotic activity. Taqman-PCR was used to quantify CDC25A^Q110del^ expression levels in 88 primary NSCLC tumor/normal tissue pairs. In patients with NSCLC, Kaplan Meier curves showed tumors expressing higher levels of CDC25A^Q110del^ relative to the adjacent lung tissues to have significantly inferior overall survival (*P* = .0018).

**Significance:**

Here we identified CDC25A^Q110del^ as a novel transcriptional variant of CDC25A in NSCLC. The sequence-specific nature of the abnormality could be a prognostic indicator in NSCLC patients as well as a candidate target for future therapeutic strategies.

## Introduction

Lung cancer remains the leading cause of malignancy-related deaths worldwide despite the advances in therapeutic modalities [1]. Non-Small cell Lung Cancer (NSCLC) is the most common type of lung cancer and is a result of accumulated molecular alterations leading to deregulation of several cellular processes including cell cycle control [2]. In NSCLC, several cell-cycle regulators that play a critical role in cell cycle check point controls are altered, which allows the cancer cells bypass different checkpoints, especially at G1/S and G2/M with subsequent uncontrolled cellular proliferation [3]–[7].

Cell division cycle 25A (CDC25A) is a member of the CDC25 family of dual specific phosphatases and plays a critical role in cell cycle progression [8], [9]. CDC25A functions to remove the inhibitory phosphates from threonine and tyrosine residues in the ATP-binding sites of CDKs, promoting cell cycle progression [10], [11]. CDC25A is also a downstream target of Chk1-mediated checkpoint pathway: activation of Chk1 by DNA damaging conditions targets CDC25A for proteasome degradation, which prevents cells with chromosomal abnormalities from progressing through the cell cycle [10], [12], [13]. While CDK1 plays a critical role for CDC25A stabilization during mitosis [8], [10], [11]. CDC25A is frequently overexpressed in cancers including NSCLC. This overexpression is associated with a more aggressive clinical behavior and inferior survival [7], [8], [12]–[19]. Though CDC25A has been extensively studied for its role in tumor progression and as a potential target for cancer treatment, the mechanisms of CDC25A overexpression in cancer remains to be investigated [12]. Some studies have shown that overexpression of CDC25A in cancers could result from post-transcriptional deregulations [20] such as overexpression of DUB3 ubiquitin hydrolase [21], inactivation of glycogen synthase kinase-3beta (GSK-3beta), which phosphorylates CDC25A to promote its proteolysis in early cell-cycle phases [22], activation of LIN28A that regulates CDC25A expression by inhibiting the biogenesis of let-7 miRNA [23], and microRNA-21 which negatively regulates CDC25A, so that its under-expression results in CDC25A overexpression in colon cancer [24].

Here we report the identification of a novel, alternatively spliced CDC25A isoform that resulted in the deletion of codon 110 termed CDC25A^Q110del^. We show that CDC25A^Q110del^ is expressed at high levels in 75% of the NSCLC cell lines. CDC25A^Q110del^ protein had higher stability and more nuclear distribution. Cells expressing high level of CDC25A^Q110del^ were more resistant to UV irradiation. In patients with NSCLC, higher CDC25A^Q110del^ levels in the tumors were associated with poor clinical outcome. Our data indicate that CDC25A^Q110del^ expression is common in NSCLC and may play a role in lung tumorigenesis and cancer progression.

## Materials and Methods

### Cell lines

HEK293 and NSCLC cells were obtained from ATCC (Manassas, VA), and maintained in DMEM - 5% fetal bovine serum. Immortalized human bronchial epithelial cell lines (HBEC), HBEC2, HBEC3, HBEC4 and HBEC5 (gift from Drs. John Minna and Jerry Shay of the University of Texas Southwestern Medical Center, Dallas, Texas) [25], were maintained in keratinocyte serum-free (KSF) media with recombinant human epidermal growth factor (rEGF) and bovine pituitary extract (Invitrogen, Carlsbad, CA). Plasmid transfection was performed using lipofectamine 2000 (Invitrogen).

### Western blotting

Cells were harvested in RIPA buffer with protease inhibitor (Roche Bioscience), and separated by SDS-PAGE. Primary antibodies against CDC25A (clones 144 and F-6), cdc2 p34 (H-297), Chk1, GAPDH (Santa Cruz Biotechnology, CA), phospho-Chk1(Ser345) (Cell Signaling Biotechnology, Danvers, MA), phospho-CDK1(Tyr15) (Calbiochem EMD chemicals Inc, Gibbstown, NJ) were used. NE-PER protein extraction kit (Pierce Biotech, Rockford, IL) were used to fractionate cytosolic and nuclear proteins. Cyclohexamide (Sigma-Aldrich, St. Louis, MO) was reconstituted in DMSO.

### RNA extraction, reverse transcription, and real-time PCR

Total RNA was extracted using TRIzol reagent, and converted to cDNA using SuperScript III First-Strand Synthesis kit (Invitrogen). Full length CDC25A cDNA was amplified using iProof High-Fidelity DNA polymerase (Bio-Rad, Hercules, CA), and cloned into pEF6/V5 His (Invitrogen, Carlsbad, CA) or pEGFP-N1(Clontech laboratories, CA) for standardization real time PCR reactions, UV radiation, CHX treatments, sequence analysis and restriction enzyme digestion, pEGFP-N1 fused to EGFP or m-Cherry was used for imaging and flowcytometry analysis or where indicated All DNA manipulations were performed in Biosafety level 1 or 2 laboratories.

To quantify the total CDC25A transcript, real-time PCR assay were assembled using the forward primer 5′-GCTCCTCCGAGTCAACAGAT -3′, reverse primer 5′- TGGACTACATCCCAACAGCTT-3′, and FAM™ dye–labeled probe 5′-ATTCTCCTGGGCCATTGGACA-3′; to quantify the wild type CDC25A transcript, the assay was assembled using the forward primer 5′ -GCTCCTCCGAGTCAACAGAT -3′, reverse primer 5′- ACTACATCCCAACAGCTTCTG- 3′ and FAM™ dye–labeled probe 5′-ATTCTCCTGGGCCATTGGACA-3′. The assay was run in triplicate with TaqMan Fast Universal PCR master mix (Applied Biosystem, Carlsbad, CA) in Applied Biosystem 7900HT Fast Real Time PCR system. In all reactions, GAPDH Fast TaqMan assay VIC dye–labeled probe was added as RNA loading control.

When the total expression of CDC25A is designated as the endogenous reference gene, the abundance of CDC25A^Q110del^ can be calculated as ΔCt = Ct _wt_−Ct _tot_. Hence, the relative abundance of CDC25^wt^ in paired tumor versus normal tissue is calculated by 2^−ΔΔCt^ method, where ΔΔCt = ΔctTumor- ΔctNormal (User Bulletin #2 Applied Biosystem). If the expression levels of CDC25A^Q110del^ and CDC25^wt^ are equal in the corresponding tumor and the adjacent normal lung tissue, the calculated relative abundance value will be 1. A value<1 indicates that the tumor expresses a higher level of CDC25A^Q110del^ than the paired normal lung tissue. Conversely, a value>1 indicates that the normal lung tissue expresses a higher level of CDC25A^Q110del^ than the paired tumor.

### Sequence analysis and restriction enzyme digestion

DNA clones were sequenced at the University of Maryland Baltimore sequencing facility or Genewiz Inc., (South Plainfield, NJ). Alignment was performed against CDC25A reference NM_001789. The cDNA clones used for the functional assays were amplified from NSCLC cell lines (Table S1). For enzymatic digestion analysis, a 292 bp fragment of CDC25A cDNA was amplified using primers: forward 5′-CACTGGAGGTGAAGAACAACAG-3′ and reverse 5′-CAGCCACGAGATACAGGTCTTA-3′, digested with the restriction endonuclease Bpu10I (New England Biolabs, Ipswich, MA) then separated on agarose gel.

### UV irradiation

For UV treatment of cultured cells, the media was removed, cells were washed twice with phosphate-buffered saline and then irradiated in uncovered tissue culture dishes with 254 nm UV light (UVC) in Stratalinker UV Crosslinker (Stratagene, La Jolla, CA). Fresh culture medium was added back, and cells were further incubated for the described time points.

### Cell cycle analysis

Cells were harvested, fixed in 70% ethanol, suspended in PI/RNase staining Buffer (BD Pharmingen™, San Jose, CA) containing 0.1% Sodium Citrate and 0.1% Triton X-100. Data analysis was done in University of Maryland Baltimore Medical Center, FlowCytometry Core and analyzed with FlowJo software.

### Imaging analysis

Cells expressing CDC25A^wt^ or CDC25A^Q110del^- fused with mCherry or EGFP were fixed in 2% paraformaldehyde, permeabilized with 0.5% tritonX-100, stained with DAPI. The Images were captured using a Zeiss LSM 510 Meta Laser Scanning Confocal Microscope with DAPI, FITC and Rhodamine fiters. The histogram representative of the FITC and rhodamine expression was produced using *image J* software.

### Cell viability assay

Cell viability was measured using the Cell Proliferation Reagent WST-1 (Roche Diagnostics Corporation, Indianapolis, IN).

### Patients and tissues

Primary NSCLC tumors and their corresponding nonmalignant adjacent lung tissues from 88 individuals with pathologic stage I to IIIa NSCLC were evaluated. All of the patients were treated with surgery alone except those with stage IIIa disease who might also had received postoperative radiation therapy and adjuvant chemotherapy, at the University of Texas M. D. Anderson Cancer Center (MDACC) from 1995 to 2000. Samples were immediately frozen and stored at −80°C. The selection of these patients was based on the availability of archived fresh tumor and corresponding normal lung tissues for the investigators. Clinical information and follow-up information for the study were based on chart review and form reports from MDACC tumor registry service. Informed consent for the use of residual resected tissues for research was obtained from all the patients enrolled in the study.

### Ethics statement

Written informed consent to use residual resected tissue for research was obtained from all patients enrolled in the study. The consent procedure and the use of these material and clinical information was reviewed and approved by University of Texas MDACC surveillance committee.

### Statistical analysis

Student *t*-test was used for statistical analysis of the functional assays. Survival analysis Kaplan Meier curves were generated with log rank analysis utilizing Proc Lifetest in SAS 9.2. All tests are two-sided and P values<.05 is considered statistically significant.

## Results

### Identification of CDC25A^Q110del^ in NSCLC

**Figure 1:**
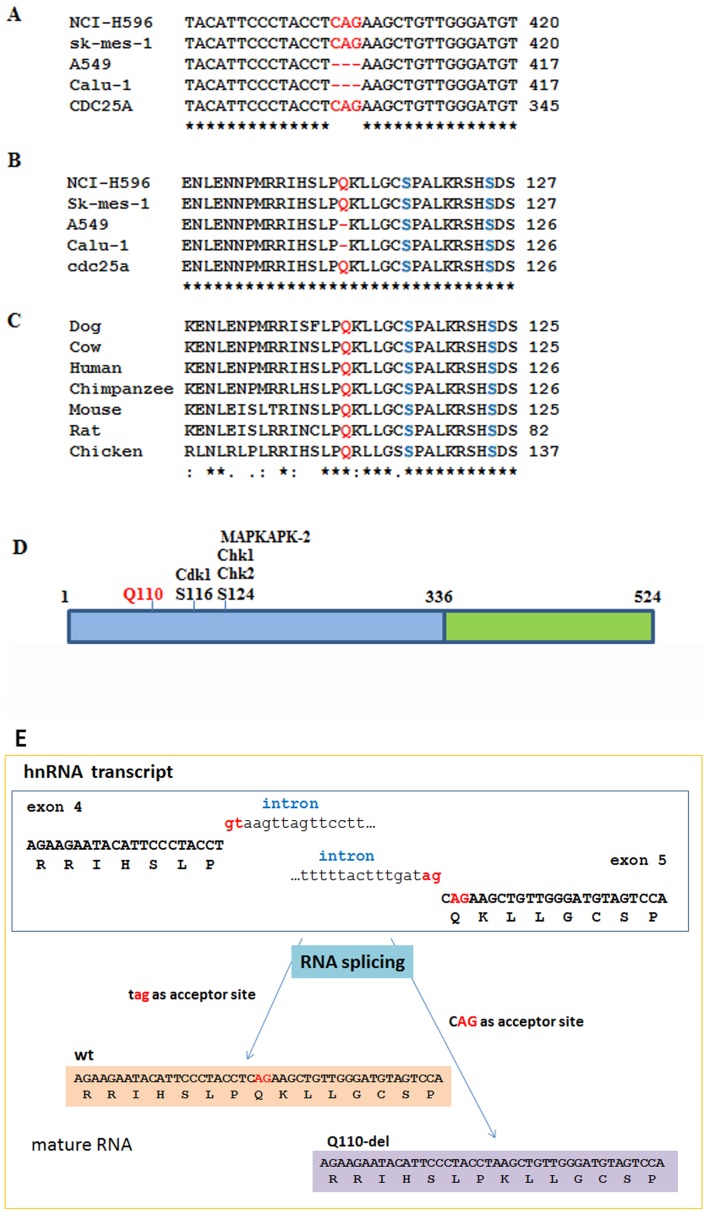
Identification of CDC25A^Q110del^ isoform in NSCLC cell lines. A. Nucleic acid sequence of triplicate deletion CDC25A-CAG (328–30) in NSCLC cell lines. B. CDC25A-CAG (328–30) translates to Q110 deletion (CDC25A^Q110del^). C. Amino acid Q110 of CDC25A is evolutionary conserved in other organisms. D. Q110 lies in the regulatory domain, closest to CDK1 mitotic stabilization phosphorylation site (S116) and Chk1 degredation phosphorylation site (S124). Red: CAG (328–30) nucleic acid deletion and corresponding amino acid Q110, shade: serine phosphorylation sites (S116 and S124). E. Schematic illustration of suggested alternative splicing site. Alternative donor site: a 5′ splice junction (donor site), changing the 3′ boundary of the upstream exon, would produce the CDC25A^wt^ transcript, while an Alternative acceptor site: a 3′ splice junction (acceptor site), changing the 5′ boundary of the downstream exon, would produce the CDC25A^Q110del^ transcript.

To investigate potential alterations of CDC25A at mRNA level, we sequenced CDC25A cDNA clones derived from a panel of 10 NSCLC cell lines. Among total 16 cDNA clones from the 10 cell lines, we observed a specific trinucleotide deletion in 7 of the 16 clones from 5 of the 10 cell lines (Fig. 1A) (Table S1). The deletion locates at positions 328–330 in reference to NM_001789.2, CDC25A transcript 1, which predicts a glutamine deletion at codon 110 (Fig. 1B). This amino acid residue is situated within the regulatory domain of CDC25A, and is conserved among several vertebrates (Fig. 1C and D). We term the novel CDC25A isoform with codon 110 deletion as CDC25A^Q110del^. This deletion is likely a result of alternative RNA splicing, since no alteration of genomic DNA sequence were found in the NSCLC cell lines (data not shown) (Fig. 1E) To confirm the presence of CDC25A^Q110del^ in NSCLC cell lines and primary NSCLC tumor tissues, we examined cDNAs from 4 NSCLC cell lines and 5 primary NSCLC tumor tissues using restriction endonuclease digestion by Bpu10I, which can cleave the sequence 5′-CCTNAGC, a unique site in CDC25A^Q110del^ sequence, to produce a shorter cleaved DNA band. All the samples showed the shorter cleaved DNA band at various densities (Fig. S1).

**Figure 2:**
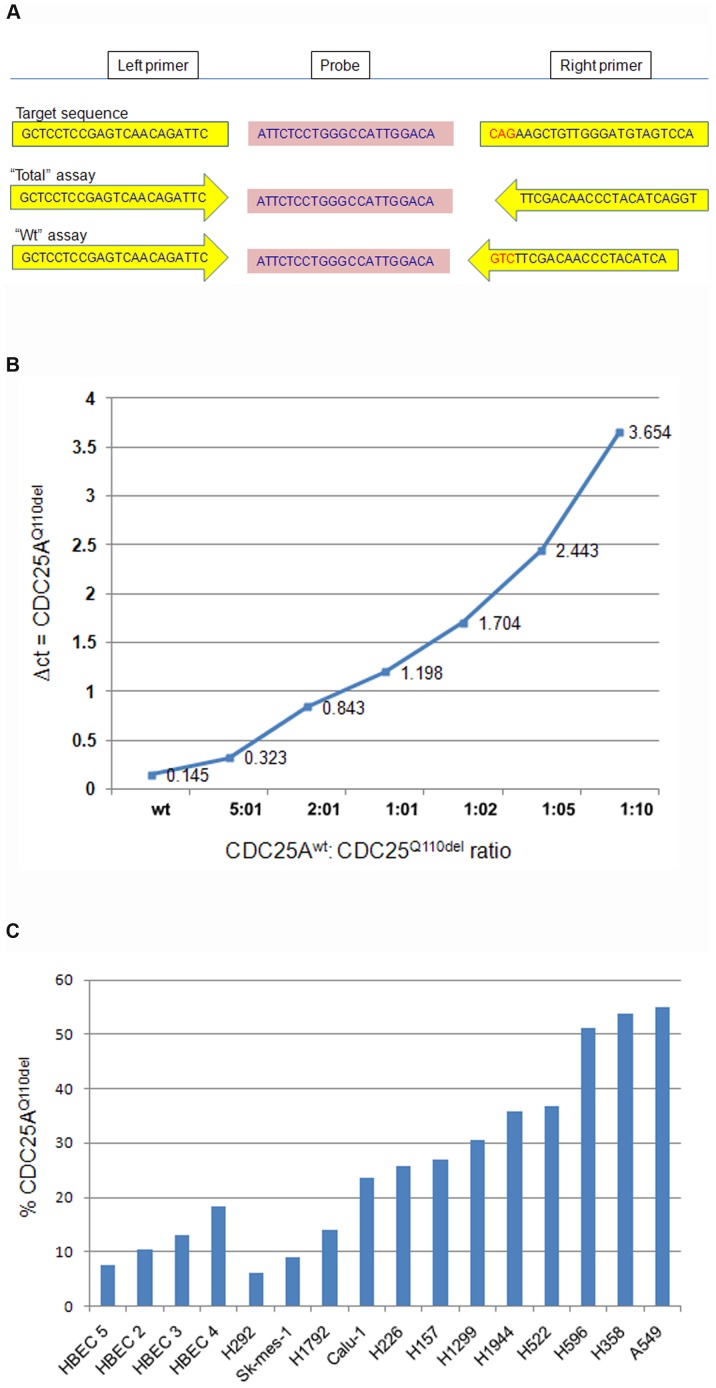
Real time-PCR quantification of CDC25A^Q110del^ in HBEC and NSCLC cell lines. A. Real-time PCR assay to assess the quantity of CDC25A^Q110del^ relative to the total CDC25A transcripts in NSCLC cell lines and tissue samples, the “Total” real time-PCR assay determines total CDC25A template in reaction (Ct_tot_) while the “wt” real time-PCR assay determines the target gene which is the CDC25A^wt^ template (Ct_wt_), then calculate the CDC25A^Q110del^ = ΔCt = (Ct_wt_−Ct_tot_). B. Standard curve illustrating Ct values corresponding to different ratios of CDC25A^wt^: CDC25A^Q110del^, pEF6-V5-His-CDC25A^wt^ and pEF6-V5-His-CDC25A^Q110del^ were incorporated together at several ratios as template in each uniplex real time PCR reaction, run in triplicates, then the CDC25A^Q110del^ calculated (CDC25A^Q110del^ = ΔCt = Ct_wt_−Ct_tot_). C. 50% of the NSCLC show CDC25A^Q110del^ values that correspond to 30–50% of total CDC25A templates in reference to the standard curve (B), while the HBEC cell lines express ∼20% of total CDC25A templates (*P* = .003).

We next devised a real-time PCR assay (Fig. 2A) to assess the quantity of CDC25A^Q110del^ among the total CDC25A transcripts in NSCLC cell lines and tissue samples, to demonstrate that the assay can quantitatively measure the relative abundance of CDC25A isoforms, we constructed a Ct curve using purified plasmid DNA containing either CDC25A^wt^ or CDC25A^Q110del^ cDNA insert. The result showed a nearly linear relationship with different wild type and Q110del ratio (Fig. 2B).This method was then used to asses CDC25A^Q110del^ expression in cell lines and tissues. In 4 HBEC cell lines, CDC25A^Q110del^ expression was detectable but at generally less than 20% of the total CDC25A transcripts (Fig. 2C). It should be noted that these cell lines were derived from immortalized human bronchial epithelial cells of patients with lung cancer. In comparison, 9 (75%) of the 12 NSCLC cell lines expressed CDC25A^Q110del^ greater than 20% of the total CDC25A transcripts including 3 (20%) of the cell lines expressed CDC25A^Q110del^ at almost 50% level (Fig. 2C). The difference of CDC25A^Q110del^ expression levels between the HBEC cell lines and the NSCLC cell lines was statistically significant (*P* = .003). Interestingly, the H596 and the H549 cell lines that show CDC25A ^Q110del^ at >50% of total CDC25A, has a high modal chromosomal number at a high percentage, NCI-H596 modal number = 71; range = 65 to 75.This is a near triploid human cell line. While A549 is a hypotriploid human cell line with the modal chromosome number of 66, occurring in 24% of cells (according to “American Type Culture Collection”). This suggests CDC25A^Q110del^ to play a role in genomic instability and cumulative malignant changes [26]. A correlation between CDC25A^Q110del^and accumulation of hyperploid cells is described below in cell cycle distribution.

### CDC25A^Q110del^ protein stability and Cell survival

**Figure 3:**
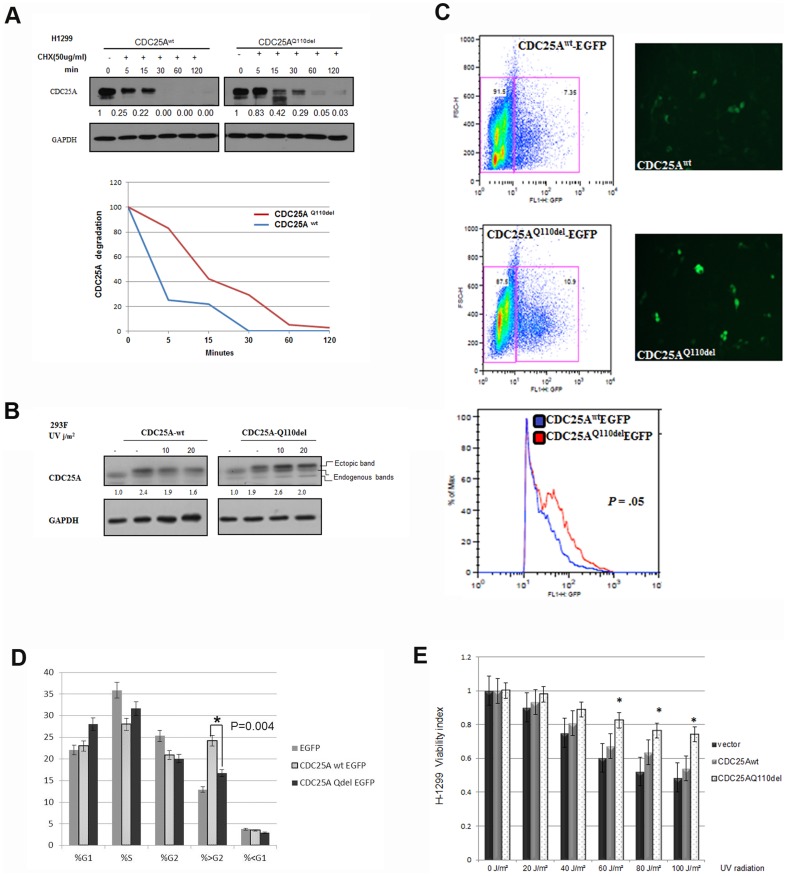
CDC25A^Q110del^ confers more CDC25A protein stability and promotes cellular survival under UV radiation. A. Time course cyclohexamide (50 ug/ml) treatment of H1299 cells transfected with pEGFPN1-CDC25A^wt^ or pEGFPN1-CDC25A^Qdel^ under unperturbed conditions showed increased half-life of CDC25A^Q110^ (∼15 minutes) versus CDC25A^wt^. B. UV radiation followed by 30 minutes incubation of 293F cells in 37°C, CDC25A^Q110del^ showed more stability compared to CDC25A^wt^. C. 293F cells plated at equal cell density and transfected with equal amount of EGFP labeled CDC25A^Q110del^ or CDC25A^wt^. After 72 hrs of transfection, flowcytometry analysis gating equal number of EGFP expressing cells for each isoform. Histogram shows intensity of expression of CDC25A^Q110del^-EGFP (mean 59.2) versus CDC25A^wt^-EGFP (mean 40.5) (t-test *P* = .05). D. The same population of cells gated for EGFP-CDC25A expression was studied for cell cycle distribution. The CDC25A^wt^-EGFP showed to arrest in the post G2 phase (>G2/M) compared to control (p = 0.055). The EGFP-CDC25A^Q110del^ expressing cells showed less accumulation of hyperploid cell population at the >G2/M phase and accelerated the cells more through mitosis compared to the EGFP-CDC25A^wt^ expressing cells (p = 0.0047). E. Cell viability assay of H1299 expressing CDC25A^wt^ versus CDC25A^Q110del^ after 24 hr of several doses of UV radiation. CDC25A^Q110del^ expression rescued H1299 sensitivity to UV radiation in relation to the control group.

CDC25A is a labile protein, tightly regulated through several phosphorylation events in its regulatory domain [8]. To investigate the effect of Q110 deletion on the fate of CDC25A protein regulation, we tested the degradation rate of CDC25A^Q110del^ using cyclohexamide (CHX) treatment. In H1299 cells treated with CHX, we observed that CDC25A^Q110del^ has a longer half-life compared to CDC25A^wt^ (Fig. 3A). Furthermore, when CDC25A^wt^ and CDC25A^Q110del^ were co-transfected, more CDC25A^Q110del^ was accumulated than CDC25A^wt^ at 72 h after transfection (Fig. S2) in both H1299 and 293F cells.

In 293F cells transfected with either CDC25A^wt^ or CDC25A^Q110del^ and treated with 10 and 20 j/m^2^ UV radiation, cells transfected with CDC25A^Q110del^ showed an enhanced protein stability 30 min after the UV irradiation but not the cells transfected with CDC25A^wt^ (Fig. 3B).

To get a more quantitative measurement of CDC25A^Q110del^ and CDC25A^wt^ levels, we measured the fluorescent intensity of CDC25A-EGFP fusion proteins gating equal number of 293F cells expressing CDC25A^wt^-EGFP or CDC25A^Q110del^-EGFP and observed a significantly higher level of fluorescent intensity in the CDC25A^Q110del^-EGFP transfected cells (Fig. 3C). The cell cycle analysis of the same gated population of cells, showed increased post G2 population (hyperploid cells) of the CDC25A^wt^-EGFP expressing cells compared to the CDC25A^Q110del^-EGFP, while the CDC25A^Q110del^-EGFP accelerated the cells more through the post G2 phase (mitosis) compared to the CDC25A^wt^ (p = 0.0047) (Fig. 3D). This suggests that the CDC25A^Q110del^ can abrogate the G2/M check point compared to the CDC25A^wt^, driving the cells more through mitosis [26], [27].

To investigate if the CDC25A^Q110del^ can affect the survival of NSCLC cells under perturbed conditions, H1299 cells transfected with CDC25A^Q110del^ were treated with UV radiation at different doses, H1299 expressing CDC25A^Q110del^ were more resistant to UV induced cell death compared to the cells transfected with the control vector or CDC25A^wt^, particularly at high UV doses (Fig. 3E).

### Cellular localization and mitotic activity of CDC25A^Q110del^

**Figure 4:**
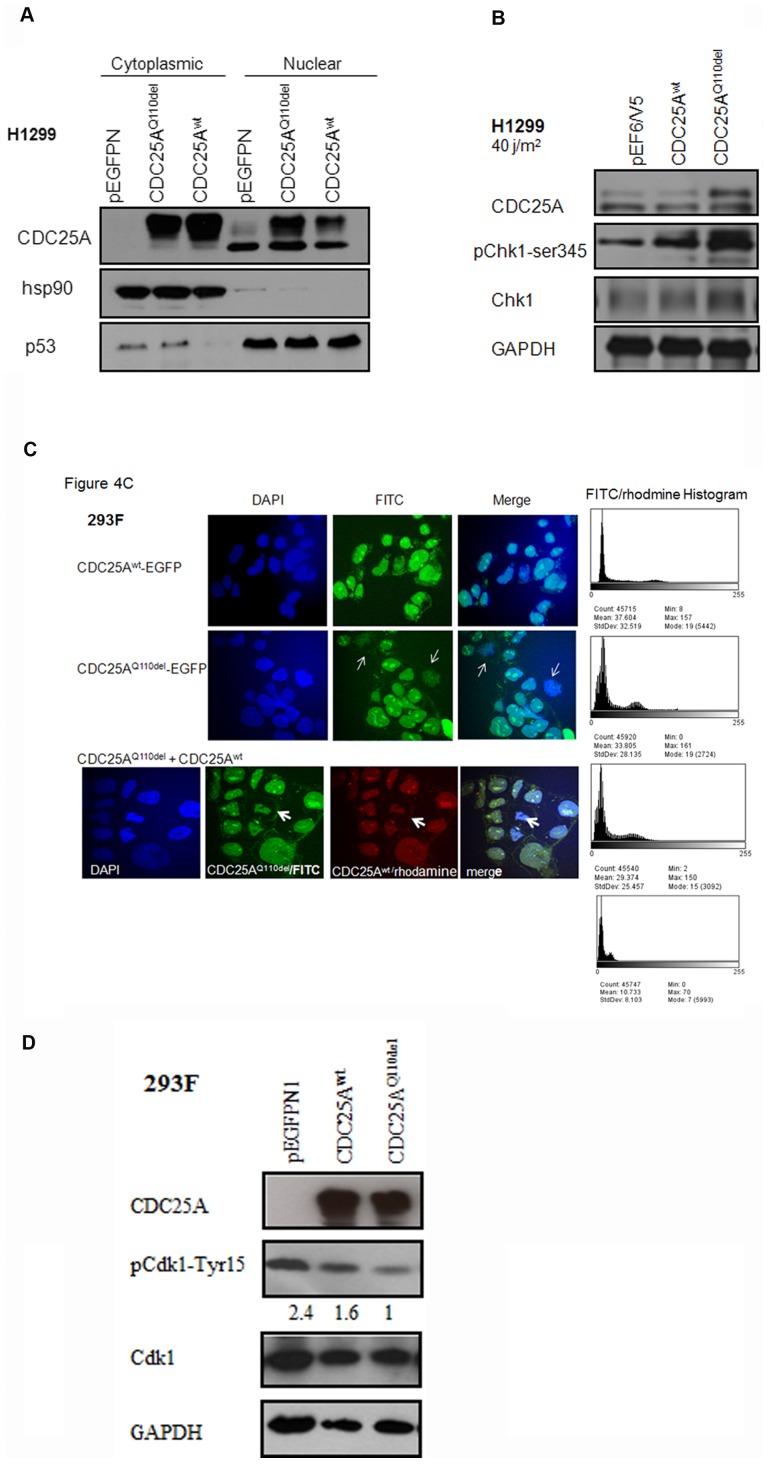
Cellular localization and mitotic activity of CDC25A^Q110del^. A. Immunoblot of H1299 cells showed CDC25A^Q110del^ to localize more in the nucleus compared to the CDC25A^wt^. B. 24 hrs after UV irradiation, CDC25A^Q110del^ showed more stability in H1299 and corresponded with more phosphorylation of DDR marker Chk1-ser345. C. Confocal Microscopy of 293F cells after 24 hr of transfection: CDC25A^Q110del^ expression showed frequent mitotic figures (metaphase “thin arrows”). Co-expression of CDC25A^wt^ and CDC25A^Q110del^ at (1∶1) ratio, mitotic activity was still noticed (cytokinesis, “bold arrows”). The histogram is representative of the pixel count for the FITC per condition and the rhodamine. D. pCDK1-Tyr15 dephosphorylation as downstream reader for relative increased phosphatase activity of CDC25A^Q110del^ compared to CDC25A^wt^ after 24 hr of transfection in 293F cells.

In H1299 cells, CDC25A^Q110del^ showed considerable increase in nuclear localization than CDC25A^wt^ (Fig. 4A). 24 hrs after UV irradiation, the cells transfected with CDC25A^Q110del^ showed higher protein stability albeit the increased phosphorylation of the upstream DNA damage response (DDR) marker pChk1-ser345 (Fig. 4B). As new evidence points CDC25A as a CDC25 family member required for full activation of nuclear CDK1 [11], [28], [29], and since Q110del is closest to S116 - the CDK1 phosphorylation site critical for stabilizing CDC25A in a feedback loop during mitosis - [11], besides our findings that showed the CDC25A^Q110del^ to drive the cells more through mitosis compared to the CDC25A^wt^ (Fig. 3D), we perused to investigate the effect of CDC25A^Q110del^ on mitotic activity and on CDK1 activation. After transfecting 293F cells with CDC25A^Q110del^-EGFP, we observed an increase in the proportion of cells in mitotic phase, a phenomenon that was not seen in the cells transfected with CDC25A^wt^-EGFP (Fig. 4C). Compared to the cells transfected with CDC25A^wt^-EGFP, the cells transfected with CDC25A^Q110del^-EGFP showed a lower level of phosphorylation at CDK1- Tyr15, 24 hrs after the transfection (Fig. 4D), which is consistent with the presence of more active CDK1 to promote G2/M phase transition (Fig. 3D). The decrease in the total CDK1 in the CDC25A^wt^ and CDC25A^Q110del^ transfected cells-compared to the control- is expected, since arrest at the cell cycle G2/M phase (Fig. 3D), can cause repression of CDK1 expression on transcriptional level in a time and cell type dependent manner [30].

### CDC25A^Q110del^expression in NSCLC and its association with clinical parameters

To determine whether there is a potential impact of CDC25A^Q110del^ expression in tumors for patients with NSCLC, we quantified CDC25A^Q110del^ expression in the primary tumors and their adjacent paired lung tissues from 88 NSCLC patients. CDC25A^Q110del^ expression ranged from undetectable to close to 100% of the total CDC25A transcripts in the tumors, with 50% of the tumors expressing CDC25A^Q110del^ in excess of 20% among the total CDC25A transcripts. Interestingly, many of the adjacent normal lung tissues from the same lung cancer patients also expressed CDC25A^Q110del^, suggesting that this is an early event in lung carcinogenesis.

**Figure 5:**
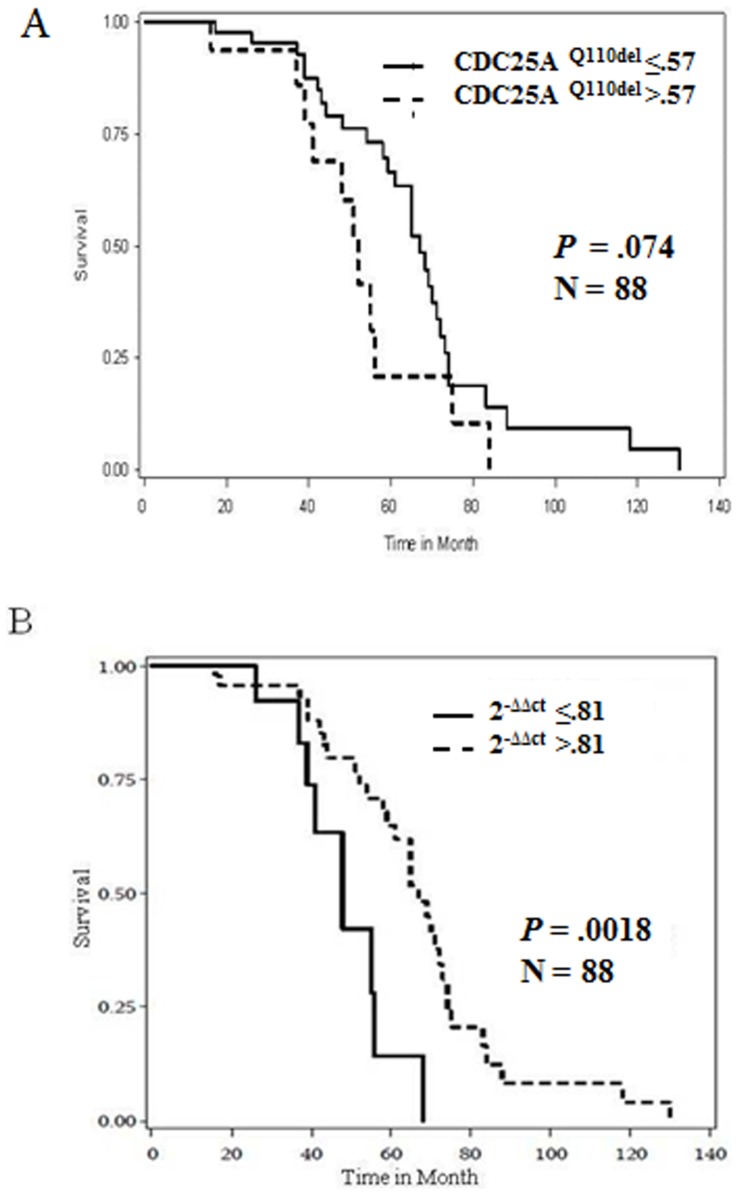
Clinical Significance of CDC25A^Q110del^. A. Kaplan Meier survival curves showed CDC25A^Q110del^ in tumor tissue to correlate with poor overall survival of NSCLC patients (log rank *P* = .074). B. Kaplan Meier Survival curves showed that when CDC25A^wt^ is higher in tumor versus normal tissue pair, it correlated with better overall survival (log rank *P* = .0018). Relative Quantification of target gene “CDC25A^wt^” in NSCLC tumor tissue relative to normal tissue pair of NSCLC patients according to the formula: 2^−ΔΔct^. CDC25A template of tumor or normal was run in triplicates of uniplex reaction for each of the Ct_wt_ and Ct_tot_ assays, and the mean was calculated for each assay.

We then analyzed the association between expression levels of CDC25A^Q110del^ in tumor tissue and clinical pathologic parameters. Except for a marginal association with adenocarcinoma histology (*P* = .068), no other association was observed (Table S2 and S3). Specifically, there was no association with stage, sex or smoking history. However, patients whose tumors had higher CDC25A^Q110del^ expression levels demonstrated a non-significant trend towards poorer overall survival (*P* = .074 by Log-rank test) (Fig. 5A; Table S2). Interestingly, when taking into consideration CDC25A^Q110del^ expression in the adjacent normal lung tissues, we observed that patients whose tumors expressed considerably higher CDC25A^Q110del^ than their paired adjacent normal lung tissues had a significantly worse overall survival (*P* = .0018 by Log-rank test) (Fig. 5B; Table S4 and S5).

## Discussion

CDC25A level is tightly regulated so that cell cycle progression and checkpoint transition is maintained in physiological conditions [20], [26], [31]–[35]. Previously, we reported that CDC25A is often over expressed in NSCLC at the transcription level [7]. In this study, we report the identification of a novel CDC25A isoform, CDC25A^Q110del^, resulting from an alternative RNA splicing. We found that CDC25A^Q110del^ was expressed in the majority of the NSCLC cell lines as well as primary tumors. In addition, the finding that histologically normal tissues adjacent to cancer frequently expressed CDC25A^Q110del^ implies that this is an early event and may play an important biological function in lung tumorigenesis.

CDC25A^Q110del^ lacks a glutamine at position 110 which is adjacent to 2 conserved serine phosphorylation sites at positions 116 and 124 (Fig. 1). S116 can be phosphorylated by Cdk1 to stabilize CDC25A in mitosis via a positive feedback loop, whereas S124 can be phosphorylated by Chk1, Chk2, and MAPKAPK-2 to accelerate CDC25A turnover [11], [14], [27], [36]. Ionizing irradiation can induce phosphorylation of S124 through Chk1 and Chk2 kinases [37], [38]. In a transgenic animal model with S124 replaced with an alanine, CDC25A was found located in centrosomes and that CDC25A levels were not reduced after ionizing irradiation [39]. In this study, we observed that CDC25A^Q110del^ has a prolonged protein half-life than CDC25A^wt^, particularly after UV irradiation, suggesting that CDC25A^Q110del^ expression may impact cellular response to the damage induced by ionizing irradiation. Consistent with this notion, we found that cells transfected with CDC25A^Q110del^ are resistant to UV induced cell death than the cells transfected with control-vector or CDC25A^wt^ (Fig. 3E), consistent with a deeper activation of Cdk1 (Fig. 4D).

CDC25A phosphatase is regulated mainly through protein degradation [8], [21], [31]. Phosphorylation of specific serine or threonine residues by protein kinases, mainly Chk1, p38 and GSK-3β, are necessary for its ubiquitin-mediated proteolysis [22], [37], [40], while phosphorylation on S18 and S116 by CDK1 can stabilize CDC25A [11]. Phosphorylation also regulates the sequestration of CDC25A by 14-3-3 protein [41]. In our study, we observed that CDC25A^Q110del^ protein has a lower turnover rate, even after UV irradiation (Fig. 3B and 4B), and more nuclear accumulation. Two possibilities may explain these observations. First, Q110 deletion in the polypeptide may produce significant conformational changes in the immediate vicinity and/or neighboring region, altering the binding affinity with its kinases, leading to a lower turnover rate of CDC25A^Q110del^. Second, CDC25A^Q110del^ protein may have a different capability to shuttle between cytoplasmic and nuclear compartments via 14-3-3 proteins [42]. The amino acid sequence immediately preceding Q110, RRIHSLP, constitute a consensus 14-3-3 binding site, (R)RxxSxP [43]. Since the binding of 14-3-3 to its target is phosphorylation dependent and is influenced by sequence context, this raises the interesting possibility that an unknown protein kinase may phosphorylate this site and influence the trafficking of CDC25A. These finding have potential significance in the context of the resistance to DNA damage in cells with ectopically expressing CDC25A^Q110del^ (Fig. 3E). Additional investigation will be needed to determine whether tumors with higher CDC25A^Q110del^ are more resistant to DNA damaging agents or radiation therapy and if targeting CDC25A^Q110del^ will sensitize these tumors to these treatments [44].

An interesting observation is the expression of CDC25A^Q110del^ in the normal appearing lung tissues adjacent to NSCLC, sometimes with high abundance, suggesting the abnormality is an early event during lung carcinogenesis [44]. Although our study cannot determine whether CDC25A^Q110del^ is also expressed in truly normal lung tissue or that its expression in the lung tissues evaluated is a result of the microscopic contamination with cancer cells from the adjacent tumor, several lines of evidence support our notion. First, 3 of the 4 immortalized human bronchial epithelial cell lines derived from patients with lung cancer expressed low level of CDC25A^Q110del^ (Fig. 2C), indicating at least some normal bronchial epithelial cells may express CDC25A^Q110del^ and its expression is independent of the presence of lung cancer cells. Second, the abundance of CDC25A^Q110del^ relative to the total CDC25A is highly variable, from undetectable to almost 100%, suggesting the expression of CDC25A^Q110del^ is determined by complicated genetic or environment factors that may also induce its expression in normal lung tissues. Third, there was no correlation of CDC25A^Q110del^ expression levels between the NSCLC tumors and the paired adjacent normal lung tissues, suggesting the expression of CDC25A^Q110del^ in the normal lung tissues was independent of the primary tumors.

However, we did observe a relationship between higher CDC25A^Q110del^ expression levels in the primary tumors and poor overall survival of the patients, particularly if the expression was significantly higher in the tumor compared to the paired adjacent lung tissue. The results suggest that CDC25A^Q110del^ is an adverse factor in lung cancer progression or treatment response. Additional studies will be required to validate the findings and further explore biological functions of CDC25A^Q110del^ in lung cancer initiation and progression.

## Supporting Information

Figure S1 **Identification of CDC25A^Q110del^ in cDNA pool of NSCLC cell lines and tumor tissue.** A. CDC25A RT-PCR product size: 292. Bpu10I restriction enzyme recognition sequence 5′-CČTNAGC-3′ flanks the deletion site in CDC25A^Q110del^ and cuts at 326 but not in the CDC25A^wt^. NEB digestion engine. B. Agarose gel shows Bpu10I digestion product of CDC25A amplified from NSCLC cell lines (lanes 2–5), and tumor tissue (lanes 8–12) using Bpu10I restriction endonuclease enzyme. Restriction fragment of CDC25A^Q110del^ versus CDC25A^wt^ clones used as control (lanes 6–7). Restriction fragment similar to that of the CDC25A^Q110del^ clone digestion was noticed in the NSCLC cell lines and tumor tissue samples.
(TIF)

Figure S2 **Increased accumulation of CDC25A^Q110del^ protein compared to CDC25A^wt^.** A. Fluorescent microscopy 72 hrs post transfection of 293F cells with CDC25A^Q110del^-mcherry versus CDC25A^wt^-mcherry showed prominent nuclear accumulation of CDC25A^Q110del^ versus CDC25A^wt^. B. H1299 72 hrs after co-transfection with CDC25A^Q110del^ and CDC25A^wt^, tagged with EGFP and mcherry fluorescent proteins alternatively. The fluorescent protein tagged to the CDC25A^Q110del^ dominated upon overlap.
(TIF)

Table S1 CDC25A cDNA clones retrieved from NSCLC cell lines.
(DOCX)

Table S2 CDC25A^Q110del^ expression in NSCLC tumor tissue and overall survival.
(DOCX)

Table S3 Tumor CDC25A^Q110del^ expression and demographic variables.
(DOCX)

Table S4 CDC25A^wt^ in NSCLC tumor versus normal tissue pair in correlation to overall patient survival.
(DOCX)

Table S5 CDC25A^wt^ in NSCLC tumor versus normal tissue pair and demographic variables.
(DOCX)
