# Supplementary figures and images for: CDC25AQ110del: A Novel Cell Division Cycle 25A Isoform Aberrantly Expressed in Non-Small Cell Lung Cancer
Source: PLoS One. 2012 Oct 5;7(10):e46464. doi: 10.1371/journal.pone.0046464 (PMC3465328; doi:10.1371/journal.pone.0046464)

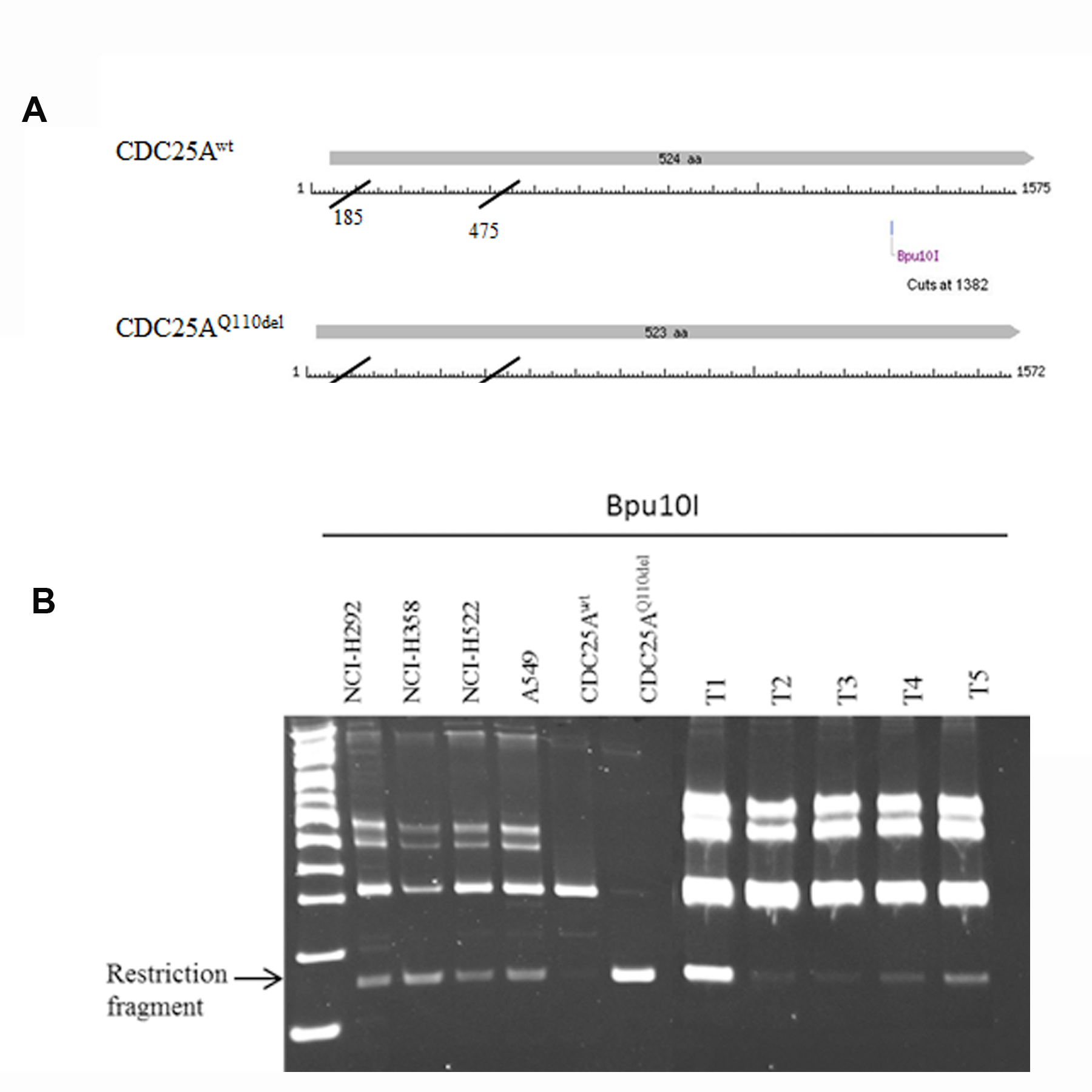

Supplement: Figure S1 — Identification of CDC25AQ110del in cDNA pool of NSCLC cell lines and tumor tissue. A. CDC25A RT-PCR product size: 292. Bpu10I restriction enzyme recognition sequence 5′-CČTNAGC-3′ flanks the deletion site in CDC25AQ110del and cuts at 326 but not in the CDC25Awt. NEB digestion engine. B. Agarose gel shows Bpu10I digestion product of CDC25A amplified from NSCLC cell lines (lanes 2–5), and tumor tissue (lanes 8–12) using Bpu10I restriction endonuclease enzyme. Restriction fragment of CDC25AQ110del versus CDC25Awt clones used as control (lanes 6–7). Restriction fragment similar to that of the CDC25AQ110del clone digestion was noticed in the NSCLC cell lines and tumor tissue samples. (TIF) [file pone.0046464.s001.tif]

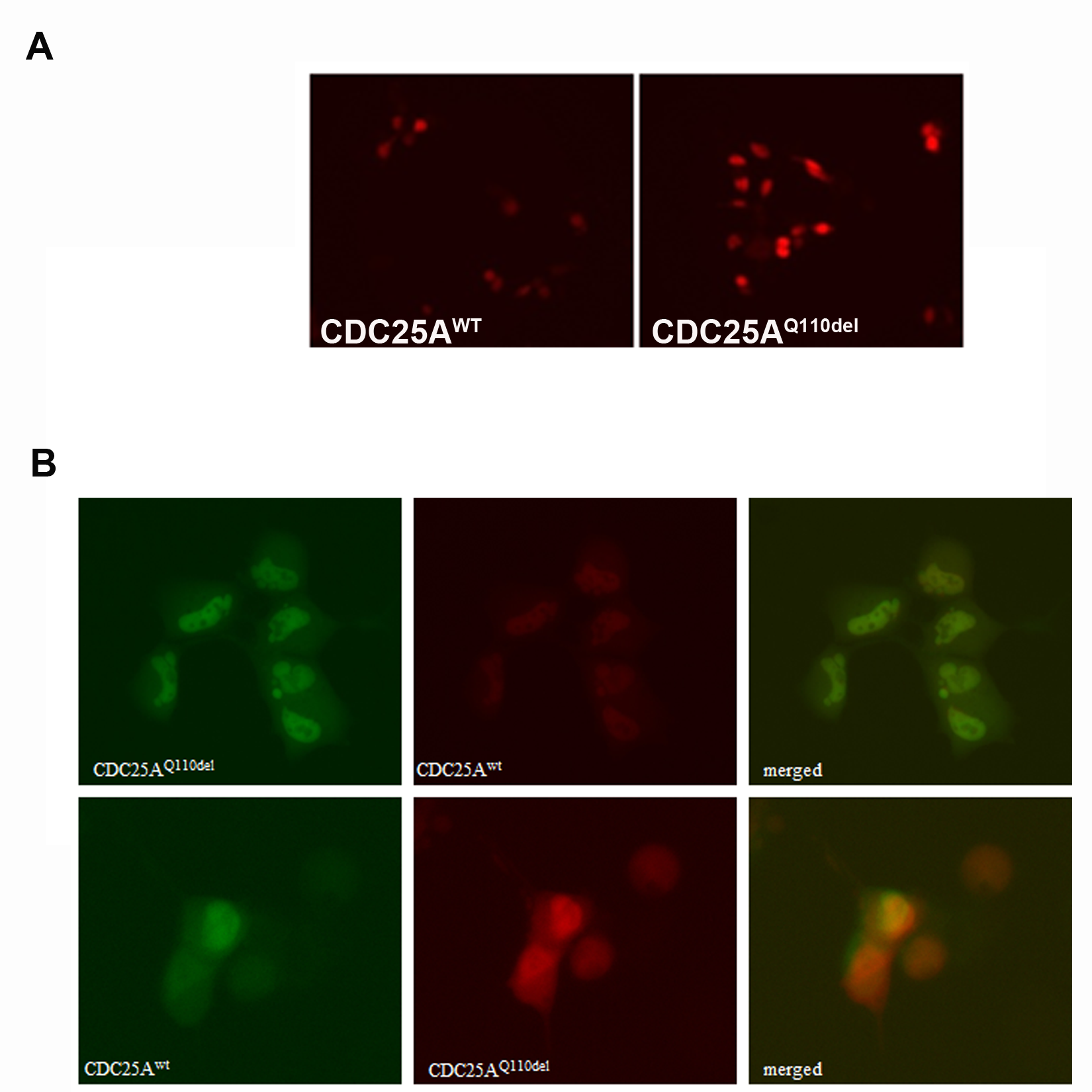

Supplement: Figure S2 — Increased accumulation of CDC25AQ110del protein compared to CDC25Awt. A. Fluorescent microscopy 72 hrs post transfection of 293F cells with CDC25AQ110del-mcherry versus CDC25Awt-mcherry showed prominent nuclear accumulation of CDC25AQ110del versus CDC25Awt. B. H1299 72 hrs after co-transfection with CDC25AQ110del and CDC25Awt, tagged with EGFP and mcherry fluorescent proteins alternatively. The fluorescent protein tagged to the CDC25AQ110del dominated upon overlap. (TIF) [file pone.0046464.s002.tif]
