# Supplementary material for: CDC25AQ110del: A Novel Cell Division Cycle 25A Isoform Aberrantly Expressed in Non-Small Cell Lung Cancer
Source: PLoS One. 2012 Oct 5;7(10):e46464. doi: 10.1371/journal.pone.0046464 (PMC3465328; doi:10.1371/journal.pone.0046464)
Supplement: Table S1 — CDC25A cDNA clones retrieved from NSCLC cell lines. (DOCX) [file pone.0046464.s003.docx]

**Table S1:** CDC25A cDNA clones retrieved from NSCLC cell lines

| **Cell Line** | **CDC25A isoforms**  **(No. of clones: sequence)** |
| --- | --- |
| NCI-H292 | 2: CDC25A^wt^ |
| NCI-H358 | 2: CDC25A^Q110del^ |
| NCI-H522 | 1: CDC25A^wt^ |
| A549 | 2: CDC25A^Q110del^ |
| NCI-H596 | 2 : CDC25A^wt^ |
| NCI-H1299 | 1: CDC25A^Q110del^ |
| NCI-H1792 | 1: CDC25A^Q110del^  1: CDC25A^wt^ |
| NCI-H1944 | 1: CDC25A^wt^ |
| calu-1 | 1 : CDC25A^Q110del^ |
| sk-mes-1 | 2 : CDC25A^wt^ |
