# Supplementary material for: CDC25AQ110del: A Novel Cell Division Cycle 25A Isoform Aberrantly Expressed in Non-Small Cell Lung Cancer
Source: PLoS One. 2012 Oct 5;7(10):e46464. doi: 10.1371/journal.pone.0046464 (PMC3465328; doi:10.1371/journal.pone.0046464)
Supplement: Table S2 — CDC25AQ110del expression in NSCLC tumor tissue and overall survival. (DOCX) [file pone.0046464.s004.docx]

**Table S2**: CDC25A^Q110del^ expression in NSCLC tumor tissue and overall survival

| **CDC25A^Q110del^** | **Tumor Tissue** | |
| --- | --- | --- |
|  | **Ct cutoff =median** | **Ct cutoff =minimizing P-value** |
| **# total Pt** | 44 (≤.44)  44 (>.44) | 63 (≤.57)  25 (>.57) |
| **# Censored (=0);**  **# Dead (=1)** | 27; 17 (≤.44)  21; 23 (>.44) | 34; 29 (≤.57)  14; 11 (>.57) |
| **P-value**  **(log-rank)** | .51 | .074 |

Proc Lifetest in SAS 9.2
