# Supplementary material for: CDC25AQ110del: A Novel Cell Division Cycle 25A Isoform Aberrantly Expressed in Non-Small Cell Lung Cancer
Source: PLoS One. 2012 Oct 5;7(10):e46464. doi: 10.1371/journal.pone.0046464 (PMC3465328; doi:10.1371/journal.pone.0046464)
Supplement: Table S3 — Tumor CDC25AQ110del expression and demographic variables. (DOCX) [file pone.0046464.s005.docx]

**Table S3:** Tumor CDC25A^Q110del^ expression and demographic variables

|  | | | | **CDC25A^Q110del^** | | **Total** | **P value** |
| --- | --- | --- | --- | --- | --- | --- | --- |
|  |  |  |  | **≤.44**  **Count (%)** | **>.44**  **Count (%)** | **Count (%)** |  |
| **Age**        **Total** | **≤63**    **>63** | | | 27(30.7)  20(22.7)  47(53.4) | 19(21.6)  22(25)  41(46.6) | 46(52.3)  42(47.7)  88(100) | .393* |
| **Pathology^$^**        **Total** | | **SCC**    **Adeno** | | 27(34.6)  15(19.2)  42(53.8) | 15(19.2) | 42(53.8)  36(46.2)  78(100) | .068* |
|  |  |  |  |  |  |  |  |
|  |  |  |  |  | 21(26.9) |  |  |
|  |  |  |  |  |  |  |  |
|  |  |  |  |  | 36(46.2) |  |  |
|  |  |  |  |  |  |  |  |
| **Stage**            **Total** | | **1**    **2**    **3** | | 26(55.3)  3(27.3)  11(57.9)  40(51.9) | 21(44.7)  8(72.7)  8(42.1)  37(48.1) | 47(61)  11(14.3)  19(24.7)  77(100) | .205** |
| **SEX**        **Total** | **male**    **female** | | 30(34.1)  17(19.3)  47(53.4) | | 20(22.7)  21(23.9)  41(46.6) | 50(56.8)  38(43.2)  88(100) | .197* |
| **Smoking** | **No** | | 17(21) | | 13(16) | 30(37) | .652* |
|  |  | |  | |  |  |  |
|  | **Yes** | | 26(32.1) | | 25(30.9) | 51(63) |  |
|  |  | |  | |  |  |  |
| **Total** |  | | 43(53.1) | | 38(46.9) | 81(100) |  |
|  |  | |  | |  |  |  |

Cutoff median (0.44) of CDC25A^Q110del^ in tumor tissue.

Clinical information for smoking available for 81 patients and clinical stage available for 77 patients only.

***** Fisher's Exact Test. **Pearson Chi-Square. ^$^ SCC: Squamous Cell Carcinoma, Adeno: Adenocarcinoma.
