# Supplementary material for: CDC25AQ110del: A Novel Cell Division Cycle 25A Isoform Aberrantly Expressed in Non-Small Cell Lung Cancer
Source: PLoS One. 2012 Oct 5;7(10):e46464. doi: 10.1371/journal.pone.0046464 (PMC3465328; doi:10.1371/journal.pone.0046464)
Supplement: Table S4 — CDC25Awt in NSCLC tumor versus normal tissue pair in correlation to overall patient survival. (DOCX) [file pone.0046464.s006.docx]

**Table S4:** CDC25A^wt^ in NSCLC tumor versus normal tissue pair in correlation to overall patient survival

|  | **CDC25A^wt^ T *v* N (2^-∆∆Ct^) ^*^** | |
| --- | --- | --- |
|  | **cutoff =median** | **cutoff =minimizing P-value** |
| **# total Pt** | 43 (≤.99)  45 (>.99) | 24 (≤.81)  64 (>.81) |
| **# Censored ; # Dead** | 23; 20 (≤.99)  25; 20 (>.99) | 15; 9 (≤.81)  33; 31 (>.81) |
| **P-value (log-rank)** | .32 | .0018 |

* CDC25A^wt^ T *v* N (2^-∆∆Ct^): CDC25A^wt^ in tumor versus normal tissue pair (User Bulletin #2 Applied Biosystem). Proc Lifetest in SAS 9.2
