# Supplementary material for: CDC25AQ110del: A Novel Cell Division Cycle 25A Isoform Aberrantly Expressed in Non-Small Cell Lung Cancer
Source: PLoS One. 2012 Oct 5;7(10):e46464. doi: 10.1371/journal.pone.0046464 (PMC3465328; doi:10.1371/journal.pone.0046464)
Supplement: Table S5 — CDC25Awt in NSCLC tumor versus normal tissue pair and demographic variables. (DOCX) [file pone.0046464.s007.docx]

**Table S5:** CDC25A^wt^ in NSCLC tumor versus normal tissue pair and demographic variables

|  | | **CDC25A^wt^ in T *v* N (2^-∆∆ct^) ^€^** | | **Total** | **P value** |
| --- | --- | --- | --- | --- | --- |
|  |  | **≤.81**  **Count (%)** | **>.81**  **Count (%)** | **Count (% )** |  |
| **Age**        **Total** | **≤63**    **>63** | 12 (13.6)  12(13.6)  24(27.3) | 34 (38.6)  30 (34.1)  64 (72.7) | 46 (52.3)  42 (47.7)  88 (100) | .815* |
| **Pathology^$^**        **Total** | **SCC**    **Adeno** | 7 (9)  14 (17.9)  21 (26.9) | 35 (44.9) | 42 (53.8)  36 (46.2)  78 (100) | .040* |
|  |  |  |  |  |  |
|  |  |  | 22 (28.2) |  |  |
|  |  |  |  |  |  |
|  |  |  | 57 (73.1) |  |  |
|  |  |  |  |  |  |
| **Stage**            **Total** | **1**    **2**    **3** | 13 (27.7)  3(27.3)  6 (31.6)  22(28.6) | 34 (72.3)  8 (72.7)  13 (68.4)  55 (71.4) | 47 (61)  11(14.3)  19 (24.7)  77 (100) | .945** |
| **SEX**        **Total** | **Male**    **Female** | 12 (13.6)  12 (13.6)  24 (27.3) | 38 (43.2)  26 (29.5)  64(72.7) | 50 (56.8)  38 (43.2)  88 (100) | .475* |
| **Smoking** | **No** | 12 (14.8) | 18 (22.2) | 30 (37) | .125* |
|  |  |  |  |  |  |
|  | **Yes** | 11 (13.6) | 40(49.4) | 51(63) |  |
|  |  |  |  |  |  |
| **Total** |  | 23 (28.4) | 58 (71.6) | 81 (100) |  |
|  |  |  |  |  |  |

^€^ CDC25A^wt^ in T *v* N: CDC25A^wt^ in tumor versus normal tissue pair (2^-∆∆Ct^: User Bulletin #2 Applied Biosystem)

* Fischer Exact test, ** Pearson Chi-Square.

^$^ SCC: Squamous Cell Carcinoma, Adeno: Adenocarcinoma,

Clinical information for smoking available for 81 patients, and clinical stage available for 77 patients only.
